# Effect of halide-mixing on the switching behaviors of organic-inorganic hybrid perovskite memory

**DOI:** 10.1038/srep43794

**Published:** 2017-03-08

**Authors:** Bohee Hwang, Chungwan Gu, Donghwa Lee, Jang-Sik Lee

**Affiliations:** 1 Department of Materials Science and Engineering, Pohang University of Science and Technology (POSTECH), Pohang, 790-784, Korea; 2 School of Materials Science and Engineering, Chonnam National University, 77 Yongbongro, Buk-gu, Gwangju, 500-757, Korea

## Abstract

Mixed halide perovskite materials are actively researched for solar cells with high efficiency. Their hysteresis which originates from the movement of defects make perovskite a candidate for resistive switching memory devices. We demonstrate the resistive switching device based on mixed-halide organic-inorganic hybrid perovskite CH_3_NH_3_PbI_3−x_Br_x_ (x = 0, 1, 2, 3). Solvent engineering is used to deposit the homogeneous CH_3_NH_3_PbI_3−x_Br_x_ layer on the indium-tin oxide-coated glass substrates. The memory device based on CH_3_NH_3_PbI_3−x_Br_x_ exhibits write endurance and long retention, which indicate reproducible and reliable memory properties. According to the increase in Br contents in CH_3_NH_3_PbI_3−x_Br_x_ the set electric field required to make the device from low resistance state to high resistance state decreases. This result is in accord with the theoretical calculation of migration barriers, that is the barrier to ionic migration in perovskites is found to be lower for Br^−^ (0.23 eV) than for I^−^ (0.29–0.30 eV). The resistive switching may be the result of halide vacancy defects and formation of conductive filaments under electric field in the mixed perovskite layer. It is observed that enhancement in operating voltage can be achieved by controlling the halide contents in the film.

Resistive switching random access memory (ReRAM) is a promising nonvolatile memory device due to its scalability, fast operation time, high density and low power consumption^1^,^2^,^3^. ReRAM stores information as two resistance states: high resistance state (HRS) and low resistance state (LRS). Numerous materials such as organics^4^,^5^, binary oxides^6^,^7^, and perovskite oxides^8^,^9^,^10^ have exhibited switchable resistance. Especially, ReRAMs based on inorganic perovskite oxide materials (e.g., Pr_0.7_Ca_0.3_MnO_3_ (PCMO)^8^, SrTiO_3_ (STO)^9^ and SrZrO_3_:Cr. (SZO:Cr)^10^) have been investigated.

Organic-inorganic perovskite materials including mixed halide perovskites are promising materials in electronic and optoelectronic devices including photodetectors^11^, light-emitting diodes^12^, and lasers^13^ in addition to solar cell applications^14^,^15^. Also, this material shows hysteresis in current-voltage responses due to defect drift or ion migration. Utilizing the defects in the organic-inorganic perovskite materials extends the application to memory devices^16^,^17^,^18^,^19^,^20^. Moreover, mixed halide perovskites have been investigated from several studies to improve the property of CH_3_NH_3_PbI_3_, such as enhancing carrier transport^21^. For example, CH_3_NH_3_PbI_3−x_Br_x_ exhibited improved carrier mobility and decreased recombination rate, and this feature can be used to fabricate low power consumption memory device due to efficient charge transport.

We selected organic-inorganic hybrid perovskite (CH_3_NH_3_PbI_3−x_Br_x_, x = 0, 1, 2, 3) to evaluate its suitability for resistive switching memory. Use of this perovskite in ReRAM is viable for three reasons. (1) CH_3_NH_3_PbI_3−x_Br_x_ exhibits hysteresis in current-voltage (I-V) curve in solar cell as a result of ion or defect migration^22^,^23^. Reaction of a charge carrier with a defect can lead to a formation of conductive filament that influences the change of the resistance state. (2) CH_3_NH_3_PbI_3−x_Br_x_ can be cast as uniform films by simple solution processing. Especially, solvent-engineering technology^24^,^25^ leads to a homogeneous and dense film. (3) The activation barrier for ionic migration is lower for Br^−^ than for I^−^26. As a consequence, this may lead to improved operating voltage and switching speed. This motivated to include Br in CH_3_NH_3_PbI_3_.

Improving operating voltage of MAPbI_3_ has been achieved by substitution of I^−^ with Br^−^, which arises from low activation barrier of Br vacancy. In this study, we evaluate CH_3_NH_3_PbI_3−x_Br_x_ as a component in nonvolatile memory devices. We also quantified how Br incorporation affects the electrical properties of different compositions of CH_3_NH_3_PbI_3−x_Br_x_ (x = 0, 1, 2, 3). The fabricated Au/CH_3_NH_3_PbI_3−x_Br_x_/ITO memory device shows low voltage operation, long data retention, and good endurance. Based on measured current electric field responses, we propose possible resistive switching mechanisms that involve migration of Br^−^ and I^−^ vacancies. We demonstrated first-principles density functional theory (DFT) calculations to clarify the lower ionic migration barrier for bromide vacancy than for iodine vacancy which leads to decreased electric field as Br content increases. Based on our present results, it is promising that the ReRAM property with CH_3_NH_3_PbI_3−x_Br_x_ can be improved by controlling the Br contents.

## Experimental Section

### Synthesis of CH_3_NH_3_I and CH_3_NH_3_Br

CH_3_NH_3_I and CH_3_NH_3_Br were synthesized from HI (57 wt% in water, Aldrich) or HBr respectively, by mixing them with CH_3_NH_2_ (40% in water, Aldrich) in 1:1 molar ratio. The reaction was performed in an ice bath under stirring for 6 h in a ventilation hood, then the solvent of the resulting solution was removed using a rotary evaporator for 1 h at 65 °C. MAI and MABr powder that precipitated during evaporation were washed with diethyl ether three times to remove residual impurities. The resulting white powder was dried in a vacuum oven, then dissolved in ethanol and recrystallized from diethyl ether. The powder was filtered using a vacuum pump then dried again in a vacuum oven^25^.

### Perovskite deposition and device fabrication

PbI_2_ and CH_3_NH_3_I were dissolved in N, N-dimethlylformamide (DMF) to obtain 30 wt% CH_3_NH_3_PbI_3_. PbBr_2_ and CH_3_NH_3_Br were dissolved in DMF to obtain 30 wt% CH_3_NH_3_PbBr_3_. The CH_3_NH_3_PbI_3−x_Br_x_ solutions were made by stoichiometric mixing 1:1 molar ratios of CH_3_NH_3_Br or CH_3_NH_3_I with PbI_2_ or PbBr_2_. The solution was stirred overnight at 70 °C under N_2_ environment. Before device fabrication, ITO/glass substrate was cleaned with isopropyl alcohol, and deionized water, then treated using UV/O_3_ (wavelength = 253.7 nm and 184.9 nm). The solution was spin coated on the ITO/glass at 7,000 rpm for 50 s. After delay time, toluene was quickly dropped onto the center of the substrate during spin coating. The obtained films were annealed at 110 °C for 15 min under N_2_ environment to eliminate residual solvents. Finally dot-shaped Au electrodes were deposited on the perovskite layer by evaporation through a shadow mask.

### Characterization

UV-vis spectrophotometer (Cary 100, Agilent Technologies) was used to characterize CH_3_NH_3_PbI_3−x_Br_x_ perovskite film. Morphological images of surface and cross section were captured using high-resolution FE-SEM (JEOL) with 10-kV acceleration voltage. Crystal structure was measured using XRD (Rigaku D/MAX-2500) with Cu Kα radiation at a step size of 0.02°. Current-voltage characteristics were measured using a Keithley 4200 in the vacuum probe station; the voltage was controlled by one of the Au electrodes under dc sweeping voltage applied as 0 V → 2 V → 0 V → −1.5 V → 0 V and the bottom electrode (ITO) was grounded.

## Results and Discussion

**Figure 1:**
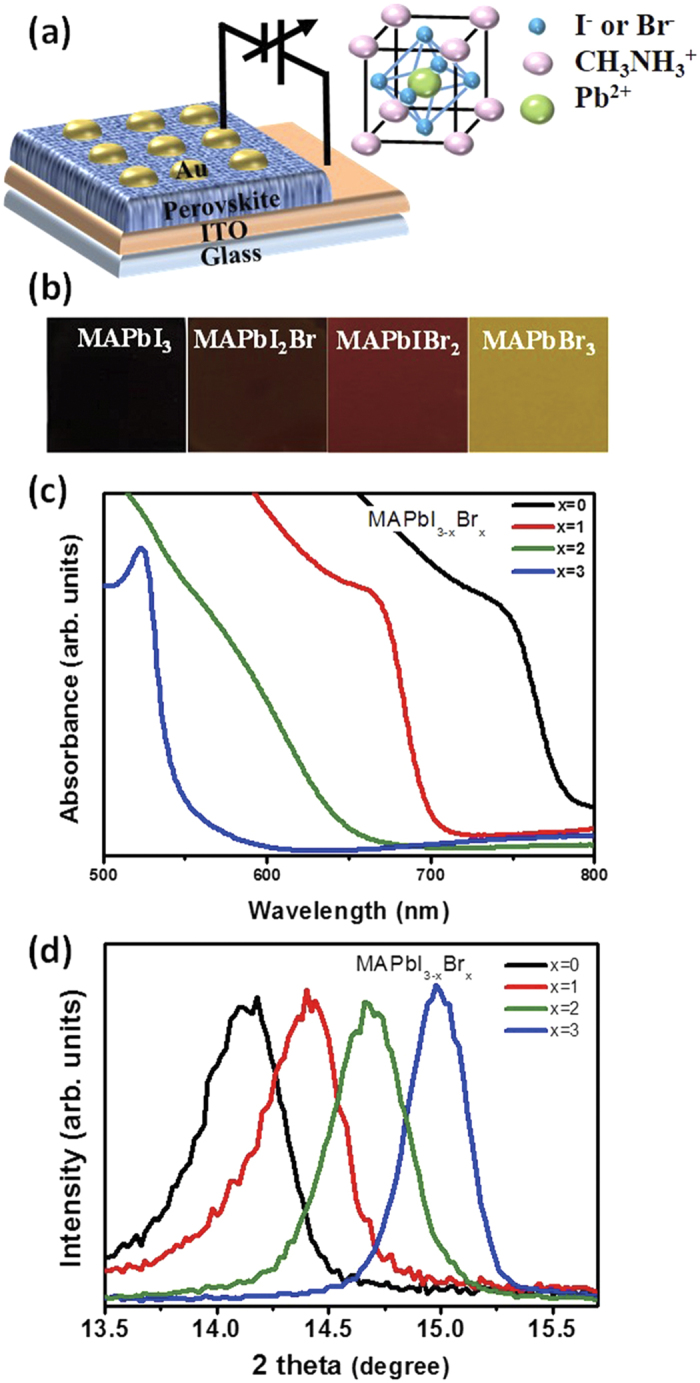
Hybrid organic-inorganic perovskite resistive switching memory devices. (**a**) Schematic diagram of memory device with a structure of Au (top electrode)/hybrid perovskite layer/ITO (bottom electrode)/glass substrate. (Right figure: schematic perovskite structure). (**b**) Photographs of CH_3_NH_3_PbI_3−x_Br_x_ films. (**c**) UV-vis absorption spectra of CH_3_NH_3_PbI_3−x_Br_x_ films. (**d**) X-ray diffraction pattern of hybrid perovskite layer with different Br^−^ ion contents to show shift of (110) peaks.

**Figure 2:**
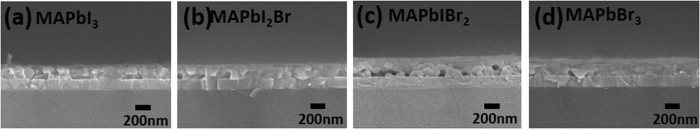
SEM images of cross-sectional views (a to d) of CH_3_NH_3_PbI_3−x_Br_x_ (x = 0, 1, 2, 3) layers.

Au/CH_3_NH_3_PbI_3−x_Br_x_/ITO-coated glass is used to demonstrate memory devices that have a metal/insulator/metal (MIM) structure. (Fig. 1a) Through the replacement of I^−^ with Br^−^, the color of the film changed from semi-transparent dark brown (CH_3_NH_3_PbI_3_) to light brown (CH_3_NH_3_PbI_2_Br, CH_3_NH_3_PbIBr_2_) then to yellow (CH_3_NH_3_PbBr_3_) with increasing Br content. (Fig. 1b). The absorbtion band edge of CH_3_NH_3_PbI_3−x_Br_x_ (x = 0, 1, 2, 3) can be tuned from a 780.20 nm wavelength (1.58 eV) to 542.82 nm wavelength (2.28 eV) (Fig. 1c). Increasing the Br content in the perovskite, the absorption band of perovskite film shifts to shorter wavelength, which indicates that energy band gap (E_g_) can be changed by the composition. The band gap values of CH_3_NH_3_PbI_3−x_Br_x_ (x = 0, 1, 2, 3) are consistent with previous reports^27^. The X-ray diffraction patterns (XRD) of CH_3_NH_3_PbI_3−x_Br_x_ (x = 0, 1, 2, 3) showed in 2θ range of 13.5–16° (Fig. 1d). The bottom XRD patterns of CH_3_NH_3_PbI_3_ exhibit peaks at 14.18°, 28.48°, and 31.96° which can be indexed to (110), (220), and (310) planes, respectively. This tetragonal structure of CH_3_NH_3_PbI_3_ indicates lattice constants with *a* = 8.855 Å and *c* = 12.659 Å calculated using the Bragg equation^28^. The top XRD patterns of CH_3_NH_3_PbBr_3_ indicated cubic perovskite phase which presented the peaks at 15°, 30.18°, and 45.92° which can be assigned to (100), (200) and (300) planes, respectively. (Figure S1a) The tetragonal phase of CH_3_NH_3_PbI_3_ remained until x = 1 and then changed to cubic phase around x = 2.^27^ As the tetragonal phase of CH_3_NH_3_PbI_3_ transited to cubic phase of CH_3_NH_3_PbBr_3_, the PbX_6_ octahedron rotated along the 〈001〉 axis which remaining connected with corner-shared octahedron, and this lead to pseudocubic lattice^27^,^29^. In CH_3_NH_3_PbI_3_, the main (110) diffraction peak of perovskite occurs at 14.18°; as Br^−^ progressively replaced I^−^ in CH_3_NH_3_PbI_3_, this diffraction peak shifted to 14.44° in CH_3_NH_3_PbI_2_Br, 14.66° in CH_3_NH_3_PbIBr_2_, and 14.98° in CH_3_NH_3_PbBr_3_. This peak shift occurs because replacing larger I atoms with smaller Br atoms decreases the lattice spacing. As the Br content increased, the tetragonal lattice parameter *a, c* decrease almost linearly (Figure S1b). The pseudocubic lattice parameter *a* was calculated, which decreased from 6.23 Å to 5.91 Å when the Br content increased. (Figure S1c)^29^ This result is in accordance with the Vegard’s law, which states that *a* varies linearly in the absence of a strong electronic effect^30^. CH_3_NH_3_PbI_3_, CH_3_NH_3_PbI_2_Br, CH_3_NH_3_PbIBr_2_, and CH_3_NH_3_PbBr_3_ deposited on ITO-coated glass substrate showed uniform layer of perovskite films which were obtained from cross-sectional SEM measurement (Fig. 2).

**Figure 3:**
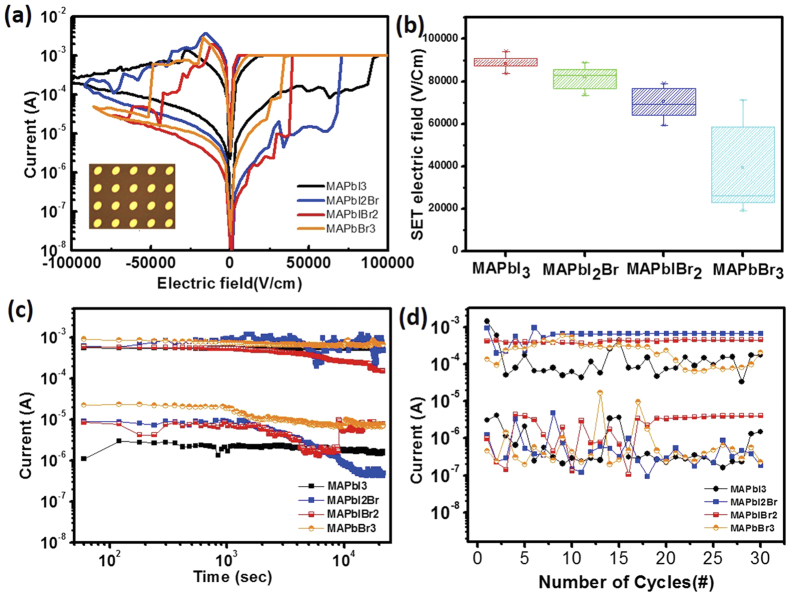
Resistive switching of Au/CH_3_NH_3_PbI_3−x_Br_x_/ITO. (**a**) I-V characteristics of Au/perovskite/ITO structure (inset: top view of memory device). (**b**) Statistical distribution of set electric fields of the hybrid perovskite resistive switching memory. (**c**) Data retention characteristics of LRS and HRS states at room temperature. (**d**) Switching endurance of perovskite memory device.

Current-Electrical field (*I- F*_*E*_) curves (Fig. 3a) in the Au/Perovskite/ITO devices exhibit bipolar resistive switching under compliance current (CC) of = 1 mA. In this work we used electric field (*F*_*E*_ = *V/t* (thickness of perovskite layers)) instead of applied bias (*V*) for comparison since there is a slight difference in thicknesses of perovskite layers with different halide composition. Ion migration depended on *F*_*E*_. During the first voltage sweep on CH_3_NH_3_PbI_3_ at positive bias from zero to set F_E_ (F_E set_ ~ 9.41 × 10^4^ V/cm), the resistance state changed from HRS (OFF state) to LRS (ON state). When a negative F_E_ was applied, the current decreased gradually at F_E_ < −2.79 × 10^4^ V/cm; the resistance changed from LRS to HRS. F_E_ at which resistance changed from HRS to LRS was lowest on CH_3_NH_3_PbBr_3_, which means that the ions or defects in CH_3_NH_3_PbBr_3_ move easily in the film.

Many types of defects (e.g., vacancies, interstitials, cation substitutions, antisite substitutions) can influence the switching behaviors in perovskites. Bromide vacancies (V^•^_Br_), lead vacancies (V’’_Pb_), and CH_3_NH_3_ vacancies (V’_MA_, where MA = methylammonium = CH_3_NH_3_) have relatively low formation energy^31^,^32^, so other interstitials or antisite defects are not likely to influence the perovskite film. Also, in a previous study, the lowest activation energy E_A_ determines the rate of vacancy migration in the perovskite film, and V^•^_Br_ has the lowest E_A_ for ionic migration^26^. The ‘set’ electric fields of devices decreased as Br content increased. (Fig. 3b) Moreover, cell-to-cell properties based on the 10 individual devices of CH_3_NH_3_PbI_3−x_Br_x_ film showed same trend such as increasing Br content leads to lower set electric field. (Figure S2) Because the V^•^_Br_ has the lowest E_A_, V^•^_Br_ may be easier to be moved to the electrode to form a conductive filament in the CH_3_NH_3_PbBr_3_ film. To confirm the conduction mechanisms of Au/CH_3_NH_3_PbI_3_ (or CH_3_NH_3_PbBr_3_)/ITO-coated glass structured ReRAM devices, a double logarithmic plot of the I-V curves is obtained (Figure S3). The conduction mechanism of the film is space-charge-limited conduction (SCLC) during HRS (I α V^2^) and filamentary type during LRS (Figure S3a,e)^33^. Intrinsic atomic defects in perovskite film, such as V^•^_I_, V’’_Pb_, V’_MA_, act as the trap sites^34^ which can be SCLC traps. Iodide vacancy which is the dominant vacancy explaining switching mechanism forms the shallow level acting as a trap site near conduction band, and these defects trap charge carriers. When the positive bias is applied, the I-V curves in the HRS region consist of two different linear regions: at low voltage (<0.3 V) the curves are linear(Ohmic conduction), while at high voltage (>0.3 V) it presents quadratic region until the set voltage was reached. (Figure S3a) At 0~0.3 V, the quantity of injected carriers is lower than that of thermally generated free charge carriers and the curve follows ohmic behavior due to partially filled traps. At high voltage (>0.3 V) during the voltage change from Ohmic to SCLC, all traps are occupied by charge carriers because of sufficient electric field, and the conduction curve obeys I α V^2^. In MAPbBr_3_ film, the logarithmic I-V curve in LRS is similar to the LRS of MAPbI_3_ that also shows ohmic conduction. (Figure S3b) Applying the bias on the MAPbBr_3_ film from 0 to 2 V changed the conduction from ohmic to SCLC in the HRS region. Through SCLC transport in I-V curves, charge trapping sites that may be formed in perovskite layer^15^ can be responsible for the resistive switching behavior of Au/perovskite/ITO device which will be explained in resistive switching mechanism.

The data retention property was evaluated to test the stability of the memory device with a reading voltage of 0.2 V at room temperature (Fig. 3c). A constant ON/OFF ratio of ~10^2^ was achieved for 2 × 10^4^ s. The current fluctuated in the HRS region but the ON/OFF ratio was maintained overall. This fluctuation is caused by charge trapping and detrapping in various trap states created by defects at different distances from the electrode^35^.

The cycling endurances of Au/perovskite/ITO devices were measured using consecutive ac voltage pulses under V_set_ = +2 V and V_reset_ = −2 V to evaluate the electrical stability (Fig. 3d). The measured voltage was 0.2 V. The endurance characteristics varied slightly over time, but neither LRS nor HRS degraded. We conclude that Au/perovskite/ITO devices are uniform and reliable. Moreover, we compared set electric field and ON/OFF ratio of our device with devices based on inorganic perovskites and organic-inorganic perovskites. Inorganic perovskites, such as V-doped SrZrO_3_ or Pr_0.7_Ca_0.3_MnO_3_, showed varied set electric field and the ON/OFF ratio was around 10^2^ or larger than 10^2^.^36^,^37^ Our device showed comparable set electric field near ~10^4^ V/cm and ON/OFF ratio (>10^2^) compared with other organic-inorganic perovskite based memory device^16^,^17^,^18^. Though larger ON/OFF ratio leads to low misreading rate with accurate controlling of the ON and OFF states, our device that shows ON/OFF ratio (>10^2^) is suitable enough to applied to memory applications.

**Figure 4:**
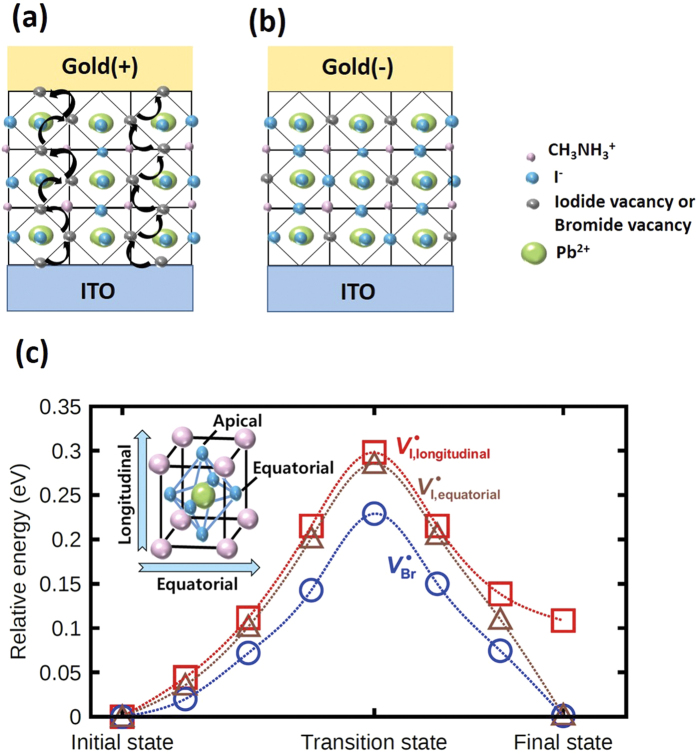
Proposed resistive switching mechanism of perovskite (CH_3_NH_3_PbI_3−x_Br_x_)-based RRAM devices. (**a**) Iodide (or Bromide) vacancy connected with top and bottom electrodes under positive bias to top electrode. (**b**) Rupture of filament under negative bias to top electrode. (**c**) Potential energy profile along two migration pathways of V^•^_I_ and one pathway of V^•^_Br_ in tetragonal CH_3_NH_3_PbI_3_ and cubic CH_3_NH_3_PbBr_3_; two energy profiles of V^•^_I_ are shown as longitudinal (red square) and equatorial (brown triangle) while one energy profile of V^•^_Br_ is shown as blue circle. Inset figure shows the schematic view of two migration pathways in tetragonal CH_3_NH_3_PbI_3_.

The hysteresis in perovskites occurs under specific scanning conditions^18^,^38^,^39^; previous studies have suggested that it is due to migration of I^−^ ions^40^,^41^ or to charge trapping^42^,^43^. The switching mechanism of Au/perovskite/ITO may be explained by defect migrations and charge trapping under the electric field (Fig. 4a,b). In order to understand the superior characteristics of CH_3_NH_3_PbI_3−x_Br_x_ with Br content, first-principles density functional theory (DFT) calculations are performed. In this study, we have chosen two compounds, CH_3_NH_3_PbI_3_ and CH_3_NH_3_PbBr_3_, which has the minimum and maximum Br context. Since the importance of the anion vacancy migration for the switching behavior in ReRAM device has been identified by previous studies^44^,^45^, we have focused our study on the migration behavior of V^•^_I_ and V^•^_Br_ in CH_3_NH_3_PbI_3_ and CH_3_NH_3_PbBr_3_ in order to clarify the decreased set electric field with increased Br content. The potential energy profile along the two migration pathways of V^•^_I_ and one migration pathways of V^•^_Br_ are shown (Fig. 4c). For tetragonal CH_3_NH_3_PbI_3_, the migration of V^•^_I_ can occur through two different pathways (longitudinal and equatorial) as shown in in-set of Fig. 4c; the longitudinal pathway is the migration between apical and equatorial positions along the long c-axis of tetragonal cell, while the equatorial pathway represents the migration between equatorial positions along xy-plane of the tetragonal cell. On the other hand, for cubic CH_3_NH_3_PbBr_3_, the migration behavior of V^•^_Br_ can show only one pattern since both pathways are identical. Substantial difference in energetic stability is observed between apical and equatorial positions for tetragonal CH_3_NH_3_PbI_3_; Our DFT calculations predict that V^•^_I_ sitting on the apical position is energetically 0.11 eV higher than that sitting on the equatorial position. (see red line in Fig. 4c) This means that V^•^_I_ prefers to place on the equatorial position and so the longitudinal migration process occurs from one equatorial position to another by passing through the apical position. As a result, the longitudinal migration accompanies two migration barriers: equatorial to apical (0.30 eV) and apical to equatorial (0.19 eV). Unlike in the case of the longitudinal migration process, the equatorial migration involves only one migration barrier of 0.29 eV between two equatorial positions. (See brown line in Fig. 4c) Thus, in tetragonal CH_3_NH_3_PbI_3_, although two migration processes have significantly different energy profiles, both have similar energy barrier (0.29~0.30 eV) for the migration of V^•^_I_. On the other hand, in cubic CH_3_NH_3_PbBr_3_, V^•^_Br_ goes through only one migration pathway; our DFT calculation predicts the energy barrier of 0.23 eV (Fig. 4c), and this calculation is in good agreement with previous studies which V^•^_Br_ (≈0.27 eV)^26^ has the lowest activation for the defect migration. Since V^•^_Br_ has lower migration barrier than V^•^_I_, it is easier to migrate to form a conductive filament. Thus, in the CH_3_NH_3_PbI_3−x_Br_x_, the decreased set electric field with the increased Br content is a result of the enhanced migration of V^•^_Br_.

Ion migration rate (*r*_*m*_) in a solid material can be estimated using the Arrhenius relation, r_m_ ∝ exp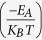, where K_B_ = 8.617 × 10^−5^ eV/K is the Boltzmann constant, and T [K] is the absolute temperature. Because V^•^_I_ has the lowest E_A_ in the CH_3_NH_3_PbI_3_^31^,^32^,^46^, the migration rate of V^•^_I_ should be large enough that defects can migrate easily in the perovskite film. Also, the jumping distance between pairs of V^•^_I_ is the shortest; this observation could explain their low E_A_. The V^•^_I_ is closer (~4.46 Å) to nearest I^−^ ions located on the edge of the PbI_6_^4−^ octaheron, than to the closest CH_3_NH_3_^+^ and Pb^2+^ ions (~6.28 Å)^47^. Because E_A_ of V^•^_I_ is low, we suggest that this is the cause of resistive switching behavior in CH_3_NH_3_PbI_3_, CH_3_NH_3_PbI_2_Br. Though CH_3_NH_3_PbI_2_Br contains V^•^_Br_, it is not sufficient to form V^•^_Br_-related conductive filament. In the pristine state without the electric field, vacancies will be spread throughout the perovskite film. Under an electric field, a positively charged V^•^_I_ migrates toward the electrode (ITO) with a negative bias during the set process. Under positive bias, V^•^_I_ will take the shortest path along the octahedral edge^46^ (Fig. 4a). Then charge carriers injected from the electrode will combine with V^•^_I_ and neutralize it.

As the applied voltage increases, a V^•^_I_ moves toward the negatively-biased electrode. Subsequently, combinations of V^•^_I_ with charge carriers will form V^•^_I_ filaments that connect the top electrode to the bottom electrode. Also, trap sites formed by Frenkel defects such as V’_MA_, V”_Pb_, and V^•^_I_^48^ will be occupied by injected electrons. Under reverse bias, electron detrapping leads to rupture of the conduction filament (Fig. 4b). In the case of CH_3_NH_3_PbBr_3_ and CH_3_NH_3_PbIBr_2,_ V^•^_Br_ would be the main cause of resistive switching properties due to the lowest E_A_ of V^•^_Br_ comparing with V^•^_I_ which was derived from DFT calculation. Moreover, in CH_3_NH_3_PbBr_3_ and CH_3_NH_3_PbIBr_2_, the migration pathway is analogous to that in CH_3_NH_3_PbI_3_.^26^ As the migration pathway of V^•^_Br_ is similar to V^•^_I_, V^•^_Br_ will form conductive filaments by combining with the charge carrier in a similar way to V^•^_Br_.

## Conclusion

We investigated organic-inorganic perovskite ReRAM based on CH_3_NH_3_PbI_3−x_Br_x_ (x = 0, 1, 2, 3) thin films as the resistive switching layer formed by solvent engineering. The memory device fabricated with CH_3_NH_3_PbBr_3_ showed the lowest ‘set’ electric field. The replacement of I with Br decreases the ‘set’ electric field, and thereby reduces the power consumption of the device. First-principles calculations show that incorporation of Br decreased the ‘set’ electrical field because compared to a V^•^_I_, a V^•^_Br_ has lower E_A_ and therefore migrates easily in perovskite films. CH_3_NH_3_PbBr_3_ perovskite ReRAM showed the lowest operation electric field of about 3.44 × 10^4^ V/cm, long data retention over 10^4^ s, and good endurance property. The resistive switching occurs by migration of V^•^_I_ and V^•^_Br_, and by formation of conducting filament under electric field. These results indicate that organic-inorganic perovskite materials have potential uses in future memory devices.

## Additional Information

**How to cite this article:** Hwang, B. *et al*. Effect of halide-mixing on the switching behaviors of organic-inorganic hybrid perovskite memory. *Sci. Rep.* **7**, 43794; doi: 10.1038/srep43794 (2017).

**Publisher's note:** Springer Nature remains neutral with regard to jurisdictional claims in published maps and institutional affiliations.

## Supplementary Material

Supplementary Information
