# Supplementary material for: Effect of halide-mixing on the switching behaviors of organic-inorganic hybrid perovskite memory
Source: Sci Rep. 2017 Mar 8;7:43794. doi: 10.1038/srep43794 (PMC5341555; doi:10.1038/srep43794)
Supplement: Supplementary Information [file srep43794-s1.doc]

*Supplementary Information*

Effect of Halide-Mixing on the Switching Behaviors of Organic-Inorganic Hybrid Perovskite Memory

Bohee Hwang,^1^ Chungwan Gu,^1^ Donghwa Lee,^2^ and Jang-Sik Lee*^1^

^1^Department of Materials Science and Engineering, Pohang University of Science and Technology (POSTECH), Pohang 790-784, Korea

^2^School of Materials Science and Engineering, Chonnam National University, 77 Yongbongro, Buk-gu Gwangju 500-757, Korea.

*Corresponding Author. E-mail: jangsik@postech.ac.kr

**Figure S1.** (a) X-ray diffraction pattern of hybrid perovskite layer with different Br^-^ ion contents. (b) The lattice parameter of tetragonal CH_3_NH_3_PbI_3-x_Br_x_ (x=0, 1, 2, 3). (c) The lattice parameter of pseudocubic or cubic CH_3_NH_3_PbI_3-x_Br_x_ (x=0, 1, 2, 3).

**Figure S2.** DC I-V curves of the Au/MAPbI_3-x_Br_x_/ITO devices obtained from 10 individual memory cells.

**Figure S3.** logI-logV plot with fitted conduction mechanism of the perovskite ReRAM in positive sweep. The experimental semilogrithmic I-V curves of (a) MAPbI_3_, and (b) MAPbBr_3_.
